# The principles of Population-Level Approaches to Dementia Risk Reduction (PLADRR)

**DOI:** 10.1371/journal.pmed.1005059

**Published:** 2026-04-28

**Authors:** Sebastian Walsh, Susanne Röhr, Joaquin Mígeot, Yuliya Bodryzlova, Etuini Ma’u, Simone Salemme, Charles R. Marshall, Timothy Daly, Gary Cheung, Blossom C. M. Stephan, Raj Kalaria, Kenneth M. Langa, Naaheed Mukadam, Leslie Grasset, Sarah Cullum, Ishtar Govia, Nikki-Anne Wilson, Daniele Urso, Ruth Peters, Jingxuan Wang, Edo Richard, Giancarlo Logroscino, Stefano Giannoni-Luza, Nicola T. Lautenschlager, Josephine E. Prynn, Cleusa P. Ferri, Susan Yates, Frank J. Wolters, Lindsay Wallace, Carol Brayne, Kaarin J. Anstey

**Affiliations:** 1 Cambridge Public Health, Hershel Smith Building, University of Cambridge, Cambridge, United Kingdom; 2 Centre for Healthy Brain Ageing (CHeBA), Discipline of Psychiatry and Mental Health, School of Clinical Medicine, Faculty of Medicine and Health, UNSW Sydney, Sydney, Australia; 3 Global Brain Health Institute (GBHI), Trinity College Dublin, Dublin, Ireland; 4 Latin American Brain Health Institute (BrainLat), Universidad Adolfo Ibañez, Santiago de Chile, Metropolitan Region of Santiago, Chile; 5 Département de médecine sociale et préventive, Université Laval, Quebec City, Canada; 6 Department of Psychological Medicine, University of Auckland, Auckland, New Zealand; 7 Te Whatu Ora Waikato, Hamilton, New Zealand; 8 Department of Biomedical, Metabolic and Neural Sciences, University of Modena and Reggio Emilia, Modena, Italy; 9 Neurology Unit, Azienda Ospedaliero-Universitaria di Modena, Modena, Italy; 10 International School of Advanced Studies, University of Camerino, Camerino, Italy; 11 Centre for Preventive Neurology, Queen Mary University of London, London, United Kingdom; 12 Bioethics Program, FLACSO Argentina, Buenos Aires, Argentina; 13 CNRS UMR 8011, Science Norms Democracy, Sorbonne Université, Paris, France; 14 Dementia Centre of Excellence, Curtin enAble Institute, Curtin University, Perth, Australia; 15 Clinical and Translational Research Institute, Newcastle University, Newcastle, United Kingdom; 16 Department of Internal Medicine and Institute for Social Research, University of Michigan, Ann Arbor, Michigan, United States of America; 17 UCL Division of Psychiatry, Maple House, London, United Kingdom; 18 Bordeaux Population Health, Université de Bordeaux & INSERM, UMR 1219, Bordeaux, France; 19 Amagi Health Ltd, London, England; 20 School of Psychology, Faculty of Science, University of New South Wales, Mathews Building, UNSW Sydney, Sydney, Australia; 21 Neuroscience Research Australia, Randwick, Australia; 22 Australian Ageing Futures Institute, University of New South Wales, Sydney, Australia; 23 Center for Neurodegenerative Diseases and the Aging Brain, Department of Clinical Research in Neurology, University of Bari ‘Aldo Moro’, Tricase, Italy; 24 The George Institute for Global Health Australia, Sydney, Australia; 25 School of Population Health, University of New South Wales, Sydney, Australia; 26 Department of Epidemiology, Boston University School of Public Health, Boston, Massachusetts, United States of America; 27 Department of Neurology, Donders Institute for Brain, Cognition and Behaviour, Radboud University Medical Centre, Nijmegen, The Netherlands; 28 Department of Public and Occupational Health, Amsterdam UMC, University of Amsterdam, Amsterdam, The Netherlands; 29 Department of Translational Biomedicine and Neuroscience (DiBraiN), University of Bari, Bari, Italy; 30 Department of Psychiatry, The University of Melbourne, Melbourne, Australia; 31 Older Adult Mental Health Program, Royal Melbourne Hospital, Melbourne, Australia; 32 Department of Population Health Sciences, King’s College London, London, United Kingdom; 33 Department of Psychiatry, Old Age Psychiatry Unit, Universidade Federal de Sao Paulo, São Paulo, Brazil; 34 Hospital Alemão Oswaldo Cruz, Social Responsibility Research Unit, São Paulo, Brazil; 35 Centre for Brain Research, University of Auckland, Auckland, New Zealand; 36 Department of Epidemiology, Erasmus MC – University Medical Center Rotterdam, Rotterdam, The Netherlands; 37 Department of Radiology & Nuclear Medicine and Alzheimer Centre, Erasmus MC – University Medical Center Rotterdam, Rotterdam, The Netherlands; 38 Department of Community Health & Epidemiology, Faculty of Medicine, Dalhousie University, Halifax, Canada

## Abstract

- Dementia is a leading health policy challenge, with cases expected to triple by 2050, particularly in low- and middle-income countries. Epidemiological evidence demonstrates falling age-specific incidence rates in high-income countries, suggesting risk can be lowered at the population level.
- The Population-Level Approaches to Dementia Risk Reduction (PLADRR) Research Group is a diverse, international network of researchers committed to investigating how structural, social, and environmental conditions can promote life course brain health and reduce dementia risk across the population.
- This Policy Forum article sets out the guiding principles of our approach, the building blocks required, our research priorities, and how PLADRR research can inform and translate into policy changes.

## Abstract 2

This Policy Forum by Sebastian Walsh and colleagues outlines the principles and aims of the Population-Level Approaches to Dementia Risk Reduction (PLADRR) research group, an international network of researchers committed to investigating how structural, social, and environmental conditions can promote brain health and reduce dementia risk across the population.

## Introduction

Dementia is a leading health policy challenge. As the global population ages, the number of people living with dementia is forecast to almost triple to over 150 million by 2050, with the majority living in low- and middle-income countries (LMICs) [1]. However, epidemiological evidence from high-income countries over the last two decades has indicated declining age-specific incidence rates [2,3], and evidence has highlighted risk and protective factors that could be amenable to risk reduction strategies [4]. This is a growing research field, with multiple lines of enquiry ongoing. Most prominent have been multidomain interventions, usually based on encouraging or facilitating lifestyle change (e.g., healthier diet, more physical activity) and sometimes inclusive of medical approaches (e.g., anti-hypertensives) in those identified at higher risk of developing dementia—with randomized controlled trial evidence suggesting these can achieve small cognitive benefits [5]. These studies are typically too short to examine an effect on dementia occurrence. Where it has been examined, there has typically been no evidence of a reduction in dementia following these individual-level interventions [5], with the possible exception of treating hypertension [6,7].

The scale of dementia, coupled with the unequal lifecourse accumulation of factors influencing risk across multiple domains, requires that research and policy approaches pay particular attention to the development of population-level strategies with broad, equitable impact [8]. However, such approaches have received inadequate research focus to date [9]. There are considerable challenges, extending from study design and causal inference methods to implementation and evaluation of policy [10]. Some of these challenges are shared with other population-level non-communicable disease (NCD) research agendas [11], whilst others are more specific to dementia, owing to its late-life nature, often decades-long pre-clinical phase, incomplete knowledge of risk factors and causal mechanisms, and the complexities of tracking changes in prevalence over time and across diverse populations [3,10,12,13]. A particular research challenge is producing valid research findings with broad relevance, whilst also recognizing the need for local tailoring of prevention strategies, owing to differing risk profiles and sociopolitical contexts [14].

The Population-Level Approaches to Dementia Risk Reduction (PLADRR) research group is an international network of researchers working together to address these challenges within and across countries and global contexts. PLADRR is a diverse, international, cross-disciplinary group with expertise across public health, epidemiology, a range of clinical specialties, health economics, ethics, and public policy. In February 2025, a delegation from PLADRR met with local clinical, research, and policy leaders in the unique socio-cultural context of Aotearoa New Zealand (see Box 1). During the meeting, delegates discussed key foundational research, evidence gaps, and future opportunities for evidence-based, population-level dementia risk reduction policy strategies. This Policy Forum article was subsequently developed to capture the complexities and nuances from these discussions, but also to map the principles and building blocks of our approach, and the next steps for this vital research field.

**Box 1. Factoring inequalities in dementia risk into dementia prevention in Aotearoa, New Zealand**

PLADRR has a global focus, whilst also recognizing the importance of tailored place-based approaches to research and policy engagement. As an example, Aotearoa, New Zealand, the location of the first PLADRR summit, presents a unique socio-cultural context, shaped by its bicultural foundations under Te Tiriti o Waitangi (Treaty of Waitangi) and growing ethnic diversity due to immigration.

As of 2023, 16% of the population was aged ≥65, with Māori (6%) and Pacific peoples (5%) underrepresented in this age group due to earlier mortality. Māori and Pacific Peoples also experience higher rates and earlier onset of age-related conditions, including dementia, yet existing services often lack cultural responsiveness [15]. Marked differences in the dementia prevention potential have been estimated between ethnic groups in Aotearoa, New Zealand, ranging from 40.8% for New Zealanders of Asian descent and 47.6% for European New Zealanders, to 50.8% for Pacific Peoples and 51.4% for Māori [16].

It is widely argued that the aforementioned inequities in New Zealand are associated with structural racism and have been attributed in part to colonialism and historical disadvantage [17–19]. Higher rates of dementia are seen in many First Nations peoples globally [20], associated with higher rates of established risk factors, including cardiometabolic conditions, head injury, and poor mental health [21]. Inequalities in dementia risk by ethnicity, as well as by socioeconomic deprivation, have also been demonstrated in countries without a history of colonization [22], demonstrating the complexity of interpreting these social trends.

Recognition of these inequalities in dementia risk has significant implications for tailoring of brain health policies to promote equity, as well as broader societal action to address deeper structural influences on health and wellbeing opportunities [23]. In New Zealand, the 2022 Pae Ora (Healthy Futures) Act centred Māori leadership in health promotion—though the dedicated Māori Health Authority created by the Act has subsequently been disestablished, speaking to the political and live nature of these debates—and emphasized community partnerships and family-centered approaches, whilst prioritizing improved health opportunities for other priority populations [24].

## What is PLADRR?

PLADRR involves changing structural, social, and environmental conditions to promote brain health and reduce dementia risk across the life course for all. The PLADRR research group aims to produce rigorous evidence-based research to support the successful translation and implementation of PLADRR into policy and practice.

**Fig 1:**
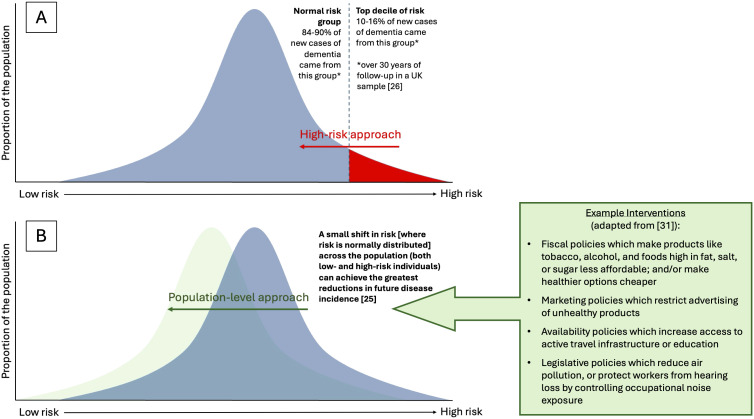
What is PLADRR?. A Population-Level Approach to Dementia Risk Reduction (PLADRR) focuses on lowering dementia risk (i.e., shifting the whole population risk distribution to the left) by a small amount (depicted on curve **B**), rather than other primary prevention approaches, for example, identifying a smaller high-risk group and working only with those individuals (depicted on curve **A**). To achieve shifting of the whole population risk distribution, PLADRR prioritizes interventions which change the social, structural, or environmental conditions that dictate what the easiest or default choice is, such that the brain-healthy option is easier for everyone to adopt, irrespective of personal resources, motivation, or agency.

Most population risk of disease is held by the large group at ‘normal risk’ rather than the smaller group at ‘high risk’ (Fig 1) [25]. For example, in a UK study, 70%–90% of dementia cases occurred in ‘normal risk’ individuals over three decades of follow-up [26]. This means that reducing risk for large swathes of the population, not just high-risk groups, is necessary to drive reductions in prevalence. This includes factors largely beyond individuals’ control, such as access to healthy foods, green space, active travel infrastructure, clean air, high-quality education, good-quality work, and freedom from poverty. Though under-researched in the past [9], there is growing evidence based on the interplay among these factors and dementia risk [23,27]. Examples of research studies that have added direct, empirical evidence to support PLADRR include natural experiment studies on the effects of historic changes in education [28] and air pollution [29] policies on cognitive aging. Further opportunities for similar studies have been hypothesized [12], for example, examining the effects of alcohol, tobacco, or obesity taxation or licensing policies on dementia incidence through interrupted time series or difference-in-difference analyses, and should be pursued as part of a broader research agenda.

Examples of population-level initiatives include social condition changes that make healthier behaviors (e.g., healthier diet, physical activity) the easier or default option. Attempting to lower population disease risk in other ways (e.g., through health education and risk awareness campaigns) can risk widening health inequalities, because conditions tend to be socially patterned such that less privileged groups have fewer resources, reduced health literacy, and/or agency to overcome barriers [30]. Changes can be achieved through large-scale policies such as excise taxation on products like tobacco and alcohol, through to smaller-scale interventions such as built environment changes to make physical activity easier and/or safer or behavioral ‘nudges’ such as increasing the prominence of healthier options in cafeterias and supermarkets [31].

PLADRR builds upon successful programs from other disease areas. For example, ‘Sure Start’, a community-based support program for young families in deprived areas within the UK, has been shown to improve educational and a range of health outcomes, including neurodevelopmental outcomes [32]. Another example, the North Karelia project, successfully reduced cardiovascular disease in a Finnish region through a large-scale public health program that included a food reformulation program to lower salt intake, broader dietary improvements in schools, hospitals, and workplaces, as well as legislative change to reduce smoking rates [33]. Applying interventions like these to PLADRR, the effects of evidence-based population-level interventions relevant to dementia can be modeled for specific effects on dementia risk, after accounting for effects on competing mortality risk [34].

## PLADRR’s mission and guiding principles

**Fig 2:**
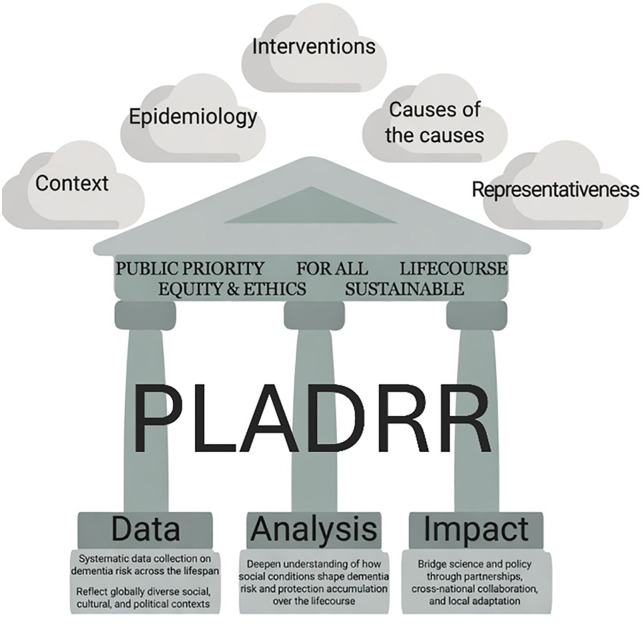
The principles, pillars, and strategic foci of PLADRR. PLADRR is based upon five principles. Dementia risk reduction should: (1) Be a public priority alongside other approaches; (2) be inclusive across society, not just those at high-risk of disease; (3) consider risk accumulation across individuals’ life courses; (4) be equitable with a focus on interventions that will narrow inequalities in dementia risk; and (5) act through action that is sustained across generations. Research activity to advance PLADRR efforts that reflects these principles will include five strategic focus areas: (i) elucidation of the relationships and interplay between structural, social, and environmental conditions (the ‘causes of the causes’) and dementia risk; (ii) the potential for mitigation of this risk by population-level interventions and policy changes, for example through natural experiment studies; (iii) studies and partnerships to explore local contexts and how they relate to brain health and inequalities; (iv) research into the temporal trends in the epidemiology of dementia risk; and (v) analysis of representative study cohorts with high generalisability to populations of interest. To enable these foci, foundational work across three pillars are required: A—efforts to ensure we have the right data on our communities available for analyses; B—developing analytic frameworks that enable careful analyses to address relevant research questions; and C—impactful communication of work through partnerships and collaborations with policymakers and community leaders to encourage translation of research into policy.

Five principles of PLADRR guide our mission, and are described below and illustrated in Fig 2:

1. **Reduce risk in the population as a public policy priority.** PLADRR should be a priority alongside the individually-focused and clinically-focused research that currently dominates the field.
2. **Reduce risk across the life course.** Strategies only focused on older people are unlikely to undo the effects of incrementally accumulated risk and protective factors for brain health across the life course (e.g., early education and mid-life cardiovascular health) [30].
3. **Reduce risk for everyone.** Shifting large sections of the population risk curve by a little will have a greater effect than intensive work with small groups at high risk (Fig 1) [26].
4. **Reduce risk equitably and ethically.** Prioritize risk reduction interventions that require little agency (individuals’ resources, effort, time, and capital) for maximum benefit (as agency is often lowest in the communities where dementia risk is highest), and ensure that risk reduction is accessible for all, including through sociocultural adaptation of interventions.
5. **Reduce risk sustainably, and cross-generationally.** Changes to structural, social, and environmental conditions can promote brain health for both current and future generations, increasing cost-effectiveness over time [30].

Guided by these principles, the research underpinning PLADRR requires strategic areas of focus (Fig 2), including elucidation of the relationships and interplay between structural, social, and environmental conditions (the ‘causes of the causes’) and dementia risk—for example, moving beyond individual dietary factors to considering the food environment [35], or beyond treatments for hearing loss to address differences in risk of hearing impairment caused by occupational noise exposure—and the potential for mitigation of risk by population-level interventions and policy changes. Alongside this, there is a need for studies to understand local contexts, how they relate to brain health, and inequalities and temporal trends in the epidemiology of dementia risk. Crucially, this research needs to be epidemiologically robust, accounting for threats to the internal and external validity of findings including the representativeness of research cohorts. These research foci are underpinned by three pillars: (1) ensuring we have the right data on our communities; (2) conducting careful analyses to answer relevant research questions; and (3) communicating research impactfully (Fig 2).

## PLADRR building blocks: From research to impact

Several enabling factors will contribute to the progress of PLADRR research. Essential to most of these is funding, although there are areas where existing resources can be repurposed or augmented to address PLADRR goals. For example, joining with other NCD campaigns and advocacy, or drawing on existing databases. All building blocks are underpinned by an equity and ethics lens. This includes recognizing data sovereignty, systemic disadvantage, and ensuring cultural sensitivity, across all stages from research design to policy implementation. The specific building blocks include:

**Datasets:** Data from cohort studies, clinical trials, administrative datasets, and biobanks can all be harnessed to extend the PLADRR evidence base. Consistent data collection over time is crucial for reliable assessment of trends in risk factor prevalence and dementia incidence. Data from other NCD research fields, increasing availability of new data linkages, and novel data collection efforts including life-course information on early-life adversity, policy exposure, and environmental conditions, can all be leveraged to accelerate PLADRR research [36].

In light of this, PLADRR aims to develop a ‘minimum dataset’ that describes the (types of) data that should be collected to inform effective, locally-tailored PLADRR research. The process to develop the minimum dataset should follow a structured consensus development process. An initial step is to review existing datasets available for PLADRR research and identification of data gaps. For example, many existing datasets come from Europe and North America, and action is needed to address data gaps particularly in LMICs, Indigenous populations, and other underrepresented groups (including within HICs) [37,38]. This will require culturally sensitive tools to reliably assess, track, and compare dementia outcomes across contexts, especially in low-literacy or culturally and linguistically diverse settings [36]. Then, insights from experts from relevant fields should be integrated, including public health, causal epidemiology, social determinants of health, and neuroscience, to develop clear guidance. Pilot work in selected sites could further enable refinement and ensure relevance to different global contexts. Novel harmonization exercises, such as the Harmonised Cognitive Assessment Protocol which includes samples from China, India, Mexico, and South Africa, will create significant opportunities for cross-country PLADRR studies [36].

**Methodology:** Several methodologies are required to advance PLADRR research and strengthen the potential for policy impact. For example, regional and cross-national analyses are required on the relationships between the exposome and dementia risk in diverse populations across the life course and across socio-economic strata. These will strengthen assessments of causality between population-level factors and dementia risk. Analyses of compression of dementia morbidity can further enhance policy arguments [39,40], while natural experiment studies can evaluate the effects of implemented policies on dementia risk [29]. Economic analyses can illustrate both the cost of inaction and the potential value of risk reduction by estimating costs of dementia to healthcare systems and through formal and informal care costs. In addition, distributional cost-effectiveness approaches can be used to examine how benefits and costs vary across socioeconomic and ethnocultural groups, and macroeconomic models can assess long-term societal gains from improved brain health [34,41]. The PLADRR research group will be direct contributors to this evidence base, as well as playing a synthesis/convening role, bringing together different types of evidence on a specific determinant of interest to identify consistencies and sources of heterogeneity by study design or context.

**Systems thinking:** Population strategies require a systems approach to identify where to most effectively target interventions and how to evaluate their full impact, including the effects on non-dementia morbidity and mortality. Systems thinking moves beyond linear cause-and-effect models, to account for the dynamic interactions and feedback loops that arise when intervening in complex real-world contexts [11]. It is possible for interventions to have unexpected impacts (positive and negative) which may be difficult to interpret without a wider understanding of the system in which they are implemented. Crucially, systems thinking acknowledges that dementia risk cannot be meaningfully reduced by interventions in isolation but requires coordinated, upstream action.

**Place:** Local, regional, national, and international locations have different cultural and ethnic compositions, different climate and economic characteristics, and exist within different regulatory and legal structures (see Box 1). Not all aspects of PLADRR will be relevant in every context, and policy priorities may be determined by understanding of specific risk profiles in a given population [42]. Understanding these unique contexts requires input from diverse sources, and tailoring measurement, analytic, and implementation approaches accordingly, in order to achieve sustainable impact. Cross-place analyses, including careful data harmonization efforts [36], can help to elucidate specific risks and policy levers that would not be readily observed otherwise [43].

**Capacity building and leadership development:** Sustainable progress requires investment in PLADRR human capital. This includes efforts to build research capacity and developing expertise in PLADRR-related methods (e.g., policy analysis, epidemiology, health economics, intervention studies, systems thinking, complex data analysis). With a particular focus on researchers from LMICs and underserved groups within HICs, PLADRR aims to support training, mentorship, exchange, and resourcing opportunities that enable researchers to lead and co-produce PLADRR-relevant data collection and analyses, publications, and policy-translation activities. Capacity building ideally extends beyond academic core skill development to include training in policy engagement, communication, interdisciplinary collaboration, open science, and ethical leadership. Regional centers of excellence, twinning programs between high- and low-resource settings, and digital platforms for knowledge exchange can further support PLADRR capacity.

**Translating research to policy through partnerships:** Translation of research evidence into policy action is not possible without multi-sectoral partnerships [10]. Key actors include government agencies, non-governmental organizations, educational and anchor institutions, public health agencies, clinicians, funders, industry partners, and community leaders [8,10]. We recognize community organizations and public representatives as essential partners, as articulated in the Consolidated Implementation Framework [44], building inclusive partnerships on shared goals, principles of trustworthiness, and empowerment.

Co-design of PLADRR studies with partners ensures that research answers the questions that are relevant to the evidence needs of decision makers and communities, and presents the evidence in a format that is accessible and impactful. For example, PLADRR members have conducted consensus exercises and qualitative studies with multidisciplinary stakeholder groups including policymakers and the public to identify key research and policy priorities for dementia risk reduction [13,45–47].

Given the overlapping nature of risk factors for many NCDs, many of which occur earlier than dementia, it may be unlikely that any specific health policy would ever explicitly be identified as a ‘PLADRR policy’. Where dementia-specific prevention policies exist, these can be assessed and influenced to ensure sufficient focus is applied to population-level strategies [48–50]. PLADRR research can also indirectly influence different aspects of the policy-making process. For example, by ensuring that the NCD prevention models used by governments to appraise preventive strategies include dementia (historically not the case), by demonstrating that doing so is feasible and can meaningfully augment the findings [34]. Through work with international organizations like the World Health Organization, PLADRR has already gained explicit recognition as an area in need of enhanced research focus [8]. This can help to ensure that the policymakers of tomorrow have the evidence they need to design impactful policies that lower the population’s dementia risk.

## Challenges to overcome

It has been increasingly recognized that dementia risk and protective factors develop across the life course [4]. Logically, dementia risk reduction policies could therefore include the whole life course within their scope [51]—creating potential ethical and research challenges.

Observational research on dementia risk factors has produced important insights, but it is difficult to fully remove the risk of residual confounding in such analyses and asserting causality is challenging [4]. Meanwhile, interventional evidence must either follow individuals up for decades, rely on the use of intermediate markers of dementia risk/brain health as an outcome, or restrict their scope to shorter-term interventions in people close to dementia onset. Longer-term studies are possible, particularly natural experiment studies of policy changes [12], but not all aspects of dementia risk will be amenable to such analyses, and statistical power can be a challenge.

A further challenge is assessing success directly through changes to dementia prevalence, requiring accurate estimates of dementia rates across populations and across time. In addition to selection and detection biases [52], assessments based on cognitive testing must carefully evaluate biases created by differing formal education levels and cultural contexts [36], whilst clinician-based assessments are expensive and must account for changes in diagnostic and clinical practices as well as public awareness over time [3]. These are surmountable challenges, but care will be needed to extend these frameworks to policy-level evidence and diverse contexts [53].

If public health programs are successfully implemented to lower the prevalence or severity of dementia risk factors, mortality may also be delayed, creating the possibility that the net effect is actually an increase in the overall number of cases of dementia and other late-life diseases [31]. Encouragement comes from studies suggesting that dementia morbidity can be compressed (rather than expanded) by positive midlife health behaviors [39,40], but these findings need further validation, ideally with inclusion of interventional/quasi-experimental evidence and health economic analyses.

A final challenge is producing outputs that promote trustworthiness between the public, policymakers, and researchers, by acknowledging these complexities of studying dementia risk reduction, whilst delivering clear, effective, and actionable messages that achieve impact [13]. Benefits from population-level action to lower dementia risk will typically not be seen within single political cycles, and policy-making processes are non-linear and rarely stem directly from evidence. Developing long-term relationships, and co-designing research studies with policymakers and the public will be key to addressing this challenge. Due to the complexity of the underlying evidence base, there will always be uncertainty in the predicted outcomes which require transparency about assumptions, sensitivity analyses to enhance robustness, and careful communication of scientific results.

## Conclusions

Dementia remains one of the most complex syndromes to study in the population. Without action, projected increases in dementia case numbers will have significant and unequal impacts on individuals, society, and the economy. Steady, incremental progress is needed to achieve success.

The PLADRR Research Group is committed to conducting impactful research on how changes to structural, social, and environmental conditions can promote life course brain health equity and reduce risk of dementia across the population and across generations. The principles and building blocks of PLADRR, outlined in this Policy Forum, will guide the maturation of this research field to maximize impact and equity. PLADRR research is built upon pillars of globally representative data, robust analysis, and impactful communication, and this paper is intended as both a call to action and a roadmap for the PLADRR research field. We are a diverse, international network of researchers, and we invite interested parties to join our community and/or to collaborate with us (contact: PLADRRResearchGroup@gmail.com). Together, we can achieve population-level reduction of dementia risk for all.

## Abbreviations:

LMICs: low- and middle-income countries
NCD: non-communicable disease
PLADRR: Population-Level Approaches to Dementia Risk Reduction
